# Applied information retrieval and multidisciplinary research: new mechanistic hypotheses in Complex Regional Pain Syndrome

**DOI:** 10.1186/1747-5333-2-2

**Published:** 2007-05-04

**Authors:** Kristina M Hettne, Marissa de Mos, Anke GJ de Bruijn, Marc Weeber, Scott Boyer, Erik M van Mulligen, Montserrat Cases, Jordi Mestres, Johan van der Lei

**Affiliations:** 1 Safety Assessment, AstraZeneca R&D Mölndal, Sweden; 2 Department of Medical Informatics, Erasmus Medical Centre, Rotterdam, The Netherlands; 3 Department of Anaesthesiology, Erasmus Medical Centre, Rotterdam, The Netherlands; 4 Chemogenomics Laboratory, Research Unit on Biomedical Informatics, Institut Municipal d'Investigació Mèdica and Universitat Pompeu Fabra, Catalonia, Spain

## Abstract

**Background:**

Collaborative efforts of physicians and basic scientists are often necessary in the investigation of complex disorders. Difficulties can arise, however, when large amounts of information need to reviewed. Advanced information retrieval can be beneficial in combining and reviewing data obtained from the various scientific fields. In this paper, a team of investigators with varying backgrounds has applied advanced information retrieval methods, in the form of text mining and entity relationship tools, to review the current literature, with the intention to generate new insights into the molecular mechanisms underlying a complex disorder. As an example of such a disorder the Complex Regional Pain Syndrome (CRPS) was chosen. CRPS is a painful and debilitating syndrome with a complex etiology that is still unraveled for a considerable part, resulting in suboptimal diagnosis and treatment.

**Results:**

A text mining based approach combined with a simple network analysis identified Nuclear Factor kappa B (NFκB) as a possible central mediator in both the initiation and progression of CRPS.

**Conclusion:**

The result shows the added value of a multidisciplinary approach combined with information retrieval in hypothesis discovery in biomedical research. The new hypothesis, which was derived in silico, provides a framework for further mechanistic studies into the underlying molecular mechanisms of CRPS and requires evaluation in clinical and epidemiological studies.

## Background

Early in the history of western medicine, the physician and the basic scientist were one and the same person. However, over the past century, clinical research developed as a separated branch from the basic sciences such as biology, molecular biology, biochemistry, and physiology. The main objective of clinical research is the collection and analysis of clinical data concerning symptoms of the disorders and responses to treatments. Based on these observations new theories about etiology and pathogenesis can be developed. However, detailed information regarding the molecular mechanisms underlying a certain disease process often remains elusive. One avenue into possible mechanisms of complex disorders is through the use of bioinformatics. Bioinformatics applies informatics techniques to organize bio-molecular data on a large scale [1].

The combination of bioinformatics and biomedical approaches is expected to result in significant advantages in both understanding mechanisms of disorders and individual susceptibility, which in turn will open many possibilities in individualized medical health care [2]. INFOBIOMED is a Network of Excellence funded by the European Union that aims at enforcing European biomedical informatics as an integrative discipline [3,4]. One of the main objectives of INFOBIOMED is to enable pilot applications in several medical fields that demonstrate the benefits of a synergetic approach in biomedical informatics. An example of such a multidisciplinary project is the use of bioinformatics tools in the investigation of the relationship between clinical and molecular data. Of course, the current literature contains information from these different domains. However, the amount of information has become so large that it is very difficult for a single individual to draw conclusions across the various disciplines. Literature based discovery support tools have been developed to bridge these interdisciplinary gaps, and novel scientific hypotheses have been generated and tested [5,6]. This approach, wherein the clinician and the basic scientist collaborate, should be beneficial in the investigation of complex disorders, where clinical research alone is not sufficient to unravel the entire disorder process.

Case studies can be useful in exploring new ways to advance multidisciplinary biomedical research. The Complex Regional Pain Syndrome (CRPS) is an example of a complex disorder from which the etiology and pathogenesis remain unelucidated for a considerable part, despite intensive research in the medical field. For this reason CRPS was chosen as a case study on how text mining techniques could be used in multidisciplinary biomedical focused research. The results should not be regarded as answers to the long unsolved questions regarding CRPS, but rather as hypothetically new insights in the molecular mechanisms underlying the disorder. The main purpose of this exercise was to assess the benefit of a new approach on hypothesis discovery, based on the use of text mining tools by a multidisciplinary team of researchers.

A brief introduction will be provided on the disorder CRPS in the next section, including a short description of the current theories about its pathogenesis.

### The Complex Regional Pain Syndrome

CRPS is a painful syndrome affecting one or more extremities of the body, marked by a wide variety of symptoms. The most prominent feature is pain, including spontaneous pain, allodynia, hyperpathia, and hyperalgesia. Additionally, the affected extremity can display changes in color and/or temperature (vasomotor disturbances), edema, alterations in transpiration, hair and nail growth (sudomotor disturbances), and muscular atrophy and/or dysfunction (motortrophic disturbances) [7,8]. It is usually described after a specific initiating event, in most cases a trauma or an operation, but sporadically it is observed after a stroke, myocardial infarction, infection or even without an obvious inciting event in a rarity of the cases [9]. The course of the disorder varies from patient to patient, but often ends in diminished function of the affected limb which impacts the quality of life of the patient. In rare cases, the disorder progresses to the point where amputation is necessary.

**Figure 1:**
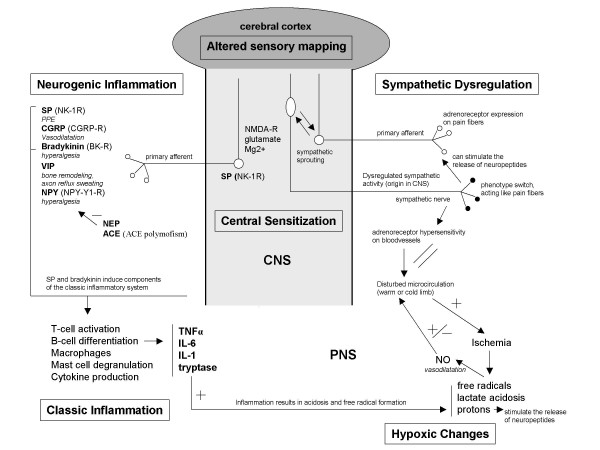
**The pathogenic mechanisms in CRPS**. The four pathogenic mechanisms in CRPS and their interactions. SP = substance P, CGRPS = Calcitonin Gene Related Protein, VIP = Vasoactive Intestinal Protein, NPY = Neuropeptide Y, NPY-Y1-R = Neuropeptide Y-Y1 receptor, NEP = Neutral Endopeptidase, ACE = Angiotensin Converting Enzyme, SMP = Sympathetically Maintained Pain, NO = nitric oxide, iNOS = inducible nitric oxidase, ONOO = peroxynitrite, NMDA-R = N-methyl-D-Aspartate receptor, NK1-R = Nuerokinin-1 receptor, CNS = central nerve system, PNS = peripheral nerve system.

The pathogenesis of CRPS evolves from disturbances in both the peripheral nervous system (PNS) and the central nervous system (CNS) (figure 1). Regarding the initial phase of the disorder, recently the interest has increased towards the role of inflammatory responses. Inflammatory signs such as swelling, redness, warmth and pain are common features in the early stage of CRPS. Classic inflammation is marked by the presence of proinflammatory cytokines and in CRPS a local increase of the cytokines TNFα, IL-6 and tryptase (a product of mast cell degranulation) was observed in blister fluid derived from the affected extremity [10,11]. IL-1 and IL-6 were also found to be increased in spinal fluid [12]. Additional to classic inflammation, a process called neurogenic inflammation has been demonstrated in CRPS [13-17]. Neurogenic inflammation resembles classic inflammation, but it is initiated by neuropeptides instead of lymphocytes and cytokines [18]. Those neuropeptides include Substance P (SP), Calcitonin Gene Related Protein (CGRP), neuropeptide Y (NPY), Bradykinin (BK) and Vasoactive Intestinal Protein (VIP). Important modulators of neurogenic inflammation are Neutral Endopeptidase (NEP) and Angiotensin Converting Enzyme (ACE) [19].

Endothelial dysfunction, hypoxic changes, and free radical damage have also been suggested as important processes in the pathogenesis of CRPS [20-24]. Ischemia, together with inflammation, can result in the formation of free radicals, lactate acidosis and altered nitric oxide (NO) synthesis. The NO metabolism in its turn has an effect on the microcirculation and thus can influence peripheral oxygen supply [25]. An impaired microcirculation might underlie a cold extremity that is observed in some cases of CRPS.

In time, the peripheral alterations give rise to disturbances at the level of the CNS, and the clinical picture of CRPS evolves more towards that of a neuropathic pain syndrome. The mechanisms behind the development of neuropathic pain have been investigated extensively in animal models [26,27]. The pathogenesis of neuropathic pain is marked by a phenomenon called neural plasticity, which is described by Woolf and Salter as the capacity of central and peripheral neurons to change their function, chemical profile, and their structure in reaction to activation of peripheral afferent nerve endings [28]. This conducts towards a state of hyperexcitability of the peripheral C- and A-fiber transducers, and of the neurons in the dorsal root ganglia (DRG) of the CNS, referred to as peripheral and central sensitization respectively. Sensitization results in a painful response to a normally not painful stimulus, leading to features as allodynia and hyperalgesia. A prominent factor in the evolvement of sensitization is the interaction between glutamate and the N-methyl-D-aspartate (NMDA) receptor, from which functional status Mg2+ is a modulator [28]. Following central sensitization, alterations at the supraspinal level might evolve, resulting in an altered sensory mapping in the cerebral cortex. This pays an additional contribution to the sensational disturbances in chronic CRPS [29].

Dysregulation of the sympathetic nerve system was classically supposed a main feature of CRPS. This was based on the observation that pain relief could be obtained by performing a sympathectomy, although only in a subset of CRPS patients. Vasomotor disturbances (warm or cold limb) [30] and sympathetically maintained pain (SMP) [31-34] are features that have been ascribed to sympathetic dysregulation. The painful responses may result from the expression of α-adrenoreceptors on sensory fibers [34]. Due to sympathetic sprouting new communication pathways are formed between sympathetic terminals and sensory neurons [35]. Additionally, the α-adrenoreceptors might have developed a hypersensitivity for normal stimulation by cathecholamines [30].

The authors have decided in this paper to focus mainly on the peripheral processes in the initial phase of CRPS. The first reason for this is that it is very feasible that the neuropathic pain component of CRPS does not form the initial pathogenesis of the disorder, but that it is preceded and sustained by the presence of the peripheral inflammatory and hypoxic reactions in the affected extremity [35], The second reason is that, until now biomolecular research on CRPS in humans and animals concerned mostly the peripheral inflammatory aspect of the disorder. Sensitization and neuronal plasticity have been studied broadly in models for neuropathic pain in general. However, they have never been demonstrated in models for CRPS in particular, although is it highly reasonable to assume that they occur in CRPS in a similar manner.

## Methods

The INFOBIOMED Network of Excellence is organized into various work packages with different aims [3]. The aim of the so called "pilot applications" is to analyse the impact of biomedical informatics in specific fields (Pharmainformatics, Genomics and Microbiology, Genomics and Chronic Inflammation, and Genomics and Cancer). The team of researchers behind the study outlined in this paper was part of the pilot application Pharmainformatics, which aims at assessing the mutual impact of BMI and pharmaceutical research. Research in this area focuses on establishing the information continuum pathology – pathway – target – ligand.

The pathogenesis of CRPS was one of the subjects that were chosen for a case study. The purpose was to investigate how to gain further insight into pathogenesis behind a complex disease and to identify possible pathways, targets and ligands for improving pharmacological therapy, using a multidisciplinary approach. The team of researchers had varying backgrounds mirroring the biomedical informatics research area (one physician with domain expertise on CRPS that was asked to participate in the work package only for this specific case study; one bioinformatician; one specialist in text mining). No formal leader was chosen for the group. All researches provided input based on their background and a plan of action took form by mutual agreement. The physician provided biological and medical concepts that are possibly linked to CRPS, the bioinformatician provided the appropriate software tool to be used in the study, and the text mining expert provided knowledge on how to best perform the extensive literature analysis. The analysis took place in September 2004, at the premises of AstraZeneca in Molndal, Sweden.

The selection of the CRPS related concepts was based on current (but not always objectified) opinions about the pathogenesis and treatment of CRPS and on different pathogenic mechanisms described in a selection of articles concerning the pathogenesis of CRPS [33,36-39]. The focus was on chemical and biochemical identities and mainly, but not completely, on the peripheral components of the disorder. The collection of concepts and their synonyms can be found in Table 1.

**Table 1:** Concepts used when building the CRPS network in PathwayAssist (description in parenthesis).

**Node Type**	**Node Name**
**Complex**	Neuronal acetylcholine receptor[16, 98]
**Functional Class**	NMDA receptor (N-methyl-D-aspartate receptor) [16, 98]
**Protein**	CALCA (calcitonin/calcitonin-related polypeptide, alpha[46, 51]), IL1A (interleukin 1 alpha) [99], IL6 (interleukin 6) [13], NGFB (nerve growth factor, beta polypeptide), NGFG (nerve growth factor, gamma subunit) [79], NPY (neuropeptide Y[100]) [10], PTGS1 (prostaglandin-endoperoxide synthase 1 (prostaglandin G/H synthase and cyclooxygenase)[101]) [10], TAC1 (tachykinin, precursor 1 (substance K, substance P, neurokinin 1, neurokinin 2, neuromedin L, neurokinin) [10], TACR1 (tachykinin receptor 1), TNF (tumor necrosis factor) 24, BDKRB2 (bradykinin receptor B2[45, 56, 57]) [102], PTGS2 (prostaglandin-endoperoxide synthase 2 (prostaglandin G/H synthase and cyclooxygenase)) [20], NTRK1 (neurotrophic tyrosine kinase, receptor, type 1) 72, TRPV1 (transient receptor potential cation channel, subfamily V, member 1), Ngfa (nerve growth factor, alpha), BDK (Bradykinin), VIP (vasoactive intestinal peptide)
**Small Molecule**	glutamate, DMSO (Dimethyl sulfoxide), PGE2 (Prostaglandin E2), magnesium, noradrenaline, capsaicin, glucocorticoid, mannitol, pentoxifylline, naproxen, bisphosphonate, verapamil, morphine, ketamine, amitriptyline, clonidine, carbamazepine, nortriptyline, lidocaine, GABA (gamma-aminobutyric acid), ketanserin, infliximab, gabapentin, amantadine, lioresal, benzodiazepine, Baclofen, N-acetyl-cystein

The text mining/entity relationship tool PathwayAssist™ (Version 2.5) was used to visualize the connections between the CRPS concepts in a network and to search for new concepts taking part within these relations. PathwayAssist is a software application developed for navigation and analysis of biological pathways, gene regulation networks and protein interaction maps. It has been used before in studies to identify genes that are involved in autism [40] and in regulation of human primordial follicle development [41]. The application has been described in more detail by Nikitin and colleagues [42]. PathwayAssist finds connections between concepts, henceforth referred to as 'nodes', by searching through a database of interactions derived from literature, using the natural language processing (NLP) based software MedScan. MedScan performs a grammatical and semantic analysis of the complete MEDLINE^® ^database of life sciences and biomedical bibliographic information. The MedScan software has been described by Novichkova et al. [43] and more recently by Daraselia et al. [44]. A brief overview of the technology is presented below.

When parsing a MEDLINE document, a semantic interpreter of the NLP component transforms the syntactic structure into a semantic structure. The syntactic structure and main constituents (surrounded by square brackets) of a sentence can be exemplified using the general sentence *Protein X inhibits protein Y*, which has the syntactic structure of [*Protein X*_[**N**]_]_ [**NP**] _[*inhibits*_[**V**] _*Protein Y*_ [**N**]_]_ [**VP**]_]_ [**S**]_. The phrasal category is shown immediately following each constituent (**NP **designates noun phrase, **N **designates noun, **V **designates verb, **VP **designates verbal phrase, **S **designates sentence). The syntactic structure will be transformed to a semantic frame of inhibition that has an 'agent' protein X and a 'patient' protein Y. The output of the semantic parse is the input for an ontological analysis that was developed by Daraselia et al. [44]. In this an 'entity' is represented either as a protein, a cellular object, a cellular process, or a small molecule and 'controls' describe functional relationships between these entities. Relations between entities are stored in a relational database. These relations can be displayed and explored through a graphical interface.

The list of concepts provided by the physician was regarded as current knowledge about CRPS (table 1) and was used as input for PathwayAssist. PathwayAssist views these concepts as nodes. The option "find only direct interactions between selected nodes" was used to search the underlying interaction database for direct connections between the CRPS concepts. Based on these connections, the system builds a network. This procedure would only detect *direct *relationships between two nodes; mechanisms that require a new intermediate node (a node not present in the original list of nodes that was provided as input) would not be detected. Such intermediate nodes, however, might represent knowledge that the scientists entering the concepts were not aware of at the time of input (such as factors acting as enhancers for a critical step in a pathway, or co-factors needed for a transcription factor to bind to DNA). Therefore, an option was used that incorporates a new node in the network if that node is the intermediate node that allows the creation of a triplet connecting two input nodes. When a new node is added to the network, all links to and from that node to all other nodes in the network are displayed.

## Results

**Figure 2:**
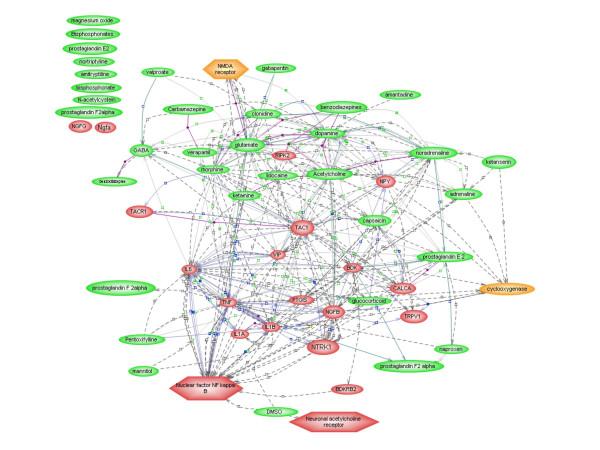
**Resulting network of CRPS concepts from PathwayAssist**. The PathwayAssist network shows that NFκB is connected with many concepts that are related to CRPS. Red circles denote proteins, red hexagons denote protein complexes, green circles denote small molecules, orange circles denote enzymes, and orange hexagons denote protein functional classes. Concepts for which PathwayAssist could not find a link to any other of the concepts in the network are shown in the upper left.

The resulting network based on the imported CRPS concepts is shown in Figure 2. In addition to the original concepts that were provided as input nodes, six new nodes have been added by the algorithm (nodes that were not in the original list of concepts known to be associated with CRPS). Of these six new nodes, the node NFκB was the one connecting to most of the original CRPS concepts and was pivotal in the final network. NFκB appeared as new node because it was part of a triple connection between mannitol and TNFα with the actual sentence being 'High glucose or mannitol also enhanced TNFalpha-stimulated NF-kappaB activity': TNFα stimulates NFκB, and mannitol acts as an enhancer of this process. The sentence describing this particular relationship between mannitol, TNFα, and NFκB had been parsed from the article by Hattori et al [45].

The transcription factor NFκB is known as a mediator in many different physiological processes [46,47]. It is also related to 7% of all the nodes that exist in PathwayAssist, which mirrors its versatility. However, even though NFκB has a very high connectivity in PathwayAssist and is involved in many physiological processes, the calculated p-value using a one-tailed Chi-square test with Yates's correction was highly statistically significant (p-value less than 0.0001). This demonstrates the significance of NFκB in the CRPS network in comparison to the whole PathwayAssist database of interactions.

To investigate the possibility that NFκB appeared in the network simply because it is a highly connected node in the database, the in PathwayAssist equally highly connected node "protein tumor protein p53" was manually included into the network and tested for its connectivity with the CRPS concepts. The p-value was found to be equally significant as for NFκB (p-value less than 0.0001). The reason for TP53 not appearing in the expanded CRPS network is related to on the algorithm that was chosen to find new nodes. In order for a new node to be incorporated into the network by PathwayAssist it has to be part of a triplet that connects two input nodes. This was the case for NFκB but not for TP53. There are also other algorithms available in PathwayAssist for incorporating new nodes in a network. These include an algorithm to find the shortest paths between the nodes in the network, an algorithm to find common targets, an algorithm to find common regulators, and also an option to expand the network by finding all nodes in the database that are connected to the nodes in the network. The shortest paths algorithm yielded 20 new nodes, the common target algorithm 46 new nodes and the common regulators algorithm yielded 38 new nodes. The option of finding all nodes connected to the nodes in the CRPS network (the expanded network algorithm) yielded 3872 nodes and it was considered to be practically infeasible to analyze all these nodes separately for their connectivity in the CRPS network. Neither NFκB nor TP53 were found by using any of the other algorithms except for the expand network algorithm. Thus, it might be possible that NFκB emerged in the CRPS network simply because it is a highly connected node in the whole PathwayAssist database. However, NFκB was still connected to more nodes in the CRPS network than the reference node TP53. Therefore it was still regarded as a candidate for a key role in the pathogenesis of CRPS

As far as the authors of this paper know, NFκB has never before been mentioned together with CRPS. For this reason NFκB was considered interesting enough to be explored further by manual research of literature, using advanced PubMed queries concerning the relation between NFκB and the CRPS concepts. Based on the results of this literature research, and on discussions about this topic within the Pharmainformatics group of the INFOBIOMED network of excellence, theories for the involvement of NFκB in the described pathogenic mechanisms in CRPS were developed. A summary of these theories is described table 2 and further explained below.

**Table 2:** Key concepts and their relations to CRPS and NFκB

**Key concept**	**Relation to CRPS**	**Relation to NFκB**
**Substance P**	Locally elevated upon electrical C-fiber stimulation [16, 98]	Induces NFκB mediated release of IL-6 and TNFα [103]
**Calcitonin Gene-related Protein**	Systemically and locally elevated [13, 16, 98]	Suppresses NFκB activity in thymic cells [104]
**Bradykinin**	Systemically elevated [13]	Activates NFκB [46, 51]
**Vasoactive Intestinal Protein**	Systemically elevated [13] Locally decreased [99]	Inhibits NFκB mediated chemokine production by macrophages [52]. Prevents NFκB binding to promoter site for NO [52]
**Neuropeptide Y**	Systemically elevated [13]	NPY-Y1-R expression is regulated by NFκB [54]
**Neutral Endopeptidase**	Speculated to be decreased [19]	No relation with NFκB described
**Angiotensin Converting Enzyme**	Speculated to be decreased [19] Polymorphism in CRPS patients [79]	Reduces NFκB activity [55]
**TNFα**	Locally increased [10]	Induced by NFκB [48], Activates NFκB [100]
**IL-1β**	Locally increased [10]	Stimulates NFκB mediated apoptosis in sympathetic neurons [101]
**IL-6**	Locally increased [10]	Induced by NFκB [49]
**Tryptase**	Locally increased [10]	Induced by NFκB [103]
**Free radicals**	Signs of free radical damage [24]	Second messenger in NFκB activity [50, 59]
**Nitric oxide**	Elevated after monocyte stimulation [102]	NO reduces NFκB activity, but ONOO induces NFκB [45, 56, 57]
**Protons (acidosis)**	Lactate increased in skin [20]	Influences NFκB activity [22, 61, 62]
*α***-receptor**	Up-regulated in analgesic skin ([72]	Pro-inflammatory responses mediated through NFκB [68-70]
**Sympathetic neuron**	Speculated to be damaged in CRPS [105]	NFκB mediates Il-1βinduced apoptosis [101]
**NMDA receptor**	Role in development of central sensitization [28].	NFκB is involved in the up-regulation of some types of NMDA receptors [63].

### Role of NFκB in CRPS related mechanisms

#### 1) NFκB involvement in neurogenic and classic inflammation

NFκB has been demonstrated to be an essential transcription factor in the mediation of the effects of neuropeptides that are also involved in CRPS: 1) SP induced expression of IL-6 and TNFα (cytokines that are locally increased in CRPS) is regulated by NFκB [48,49]; 2) Glutamate, released in the dorsal horn together with SP induces neuronal apoptosis through NFκB induction [50]; 3) Expression of CGRP (an important neuropeptide in CRPS) is induced by an NFκB mediated pathway, initiated by IL-1β stimulation [12]; 4) Bradykinin activates NFκB and cyclooxygenase-2 via more than one pathway [46,51]; 5) VIP inhibits NFκB mediated chemokine production by macrophages [52]. It also prevents NFκB from binding to the promotor site for nitric oxide synthethase iNOS, thereby affecting the microcirculation in CRPS [53]; 6) For the NPY receptor, potential NFκB binding sites have been found in the promoter regions, suggesting a role for NFκB in the expressional regulation of this receptor [54]; 7) The important inflammatory modulator ACE reduces NFκB activity [55]. The overall effect appears to be that NFκB activity is upregulated by the neuropeptides involved in CRPS, resulting in a pro-inflammatory response mediated through NFκB.

#### 2) NFκB involvement in hypoxic changes

NFκB activity is inhibited by NO [45,56,57]. However, peroxynitrite (ONOO), which is formed from NO after reaction with radical oxygen intermittents, sustains NFκB activity [45]. NFκB also is involved in the expression of inducible nitric oxide synthethase (iNOS) induced by pro-inflammatory cytokines TNFα and IL1-β, which are locally elevated in CRPS [10,58]. Additionally, NFκB activity is involved in and affected by free radical formation [50,59,60] and acidosis [22,61,62].

#### 3) NFκB and neuropathic pain

Several animal models demonstrate the role of NFκB in pain induction and pain maintenance in the CNS. For example, NFκB is involved in the upregulation of some types of NMDA receptors which are important mediators in the development of central sensitization [63]. Additionally, NFκB in the CNS mediates IL-1 induced COX-2 up-regulation and prodynorphin expression [64,65]. The derived compound dynorphin is an endogenous opiate that causes hyperalgesia and allodynia in mice. In line with this, intrathecal (in the central spinal fluid) or neuronal injected NFκB inhibitors attenuate proinflammatory cytokine mediated pain in rats [66,67].

#### 4) NFκB involvement in sympathetic dysregulation

Pro-inflammatory responses induced by the catecholamines derived from the sympathetic nervous system, which might be dysregulated in CRPS, are mediated by NFκB [68-70]. Additionally, NFκB up-regulates directly the expression of the β-adrenoreceptor on immune cells [71]. Moreover, the expression of the α-receptor might be regulated by NFκB in an indirect way which involves Il-1β mediated apoptosis of sympathetic neurons, leading to up-regulation and/or hypersensitivity of the peripheral α-receptors expressed on blood vessels, immune cells, and nociceptive primary afferents [72].

## Discussion

This paper describes how a multidisciplinary team of investigators applied advanced information retrieval methods, in the form of a text mining/entity relationship tool, with the purpose of discovering new hypotheses concerning the pathogenesis of a complex disorder, exemplified here by CRPS. The exercise should be regarded as a "journey" to discover what benefit could emerge from this kind of collaboration between bioinformatics experts and clinical experts. The purpose was neither to assess the specificity of the used methodology, nor to discover complete underlying biochemical pathways.

The text mining/entity relationship tool PathwayAssist provided a new concept, named NFκB, which is related to a majority of the concepts described to be involved in CRPS. After manual literature search, NFκB appeared to be a link between the various, previously described pathogenic mechanisms and appeared to serve as a mediating component in their connections.

### Validity of the method

The MedScan system has been manually validated by the developers through random extraction and analysis of direct physical protein-protein interactions from MEDLINE [44]. From the interactions extracted by MedScan, 91% was correct (precision). Most errors were attributed to the extracted functional interference between proteins rather than physical interaction. Coverage (recall), however, was low: 21%. This is primarily due to the low coverage rate of the NLP component (34%). The concept network on CRPS (figure 2) was validated manually by the authors of this paper by checking all relations leading to or from NFκB. Out of 38 relations, 34 were correct and 4 incorrect.

Naturally, it would be helpful if some sort of independent relevance score could be provided for new hypotheses generated by the method used in this report. In this view, a p-value was calculated for the new node NFκB in the network using a one-tailed Chi-square test with Yates's correction, comparing its node connectivity in the PathwayAssist database to its node connectivity in the CRPS concepts network. In addition, the protein TP53 was chosen as a reference case because of the equally high connectivity in the PathwayAssist database compared to NFκB. Unfortunately, TP53 was found to be equally significant in the CRPS network as NFκB. However, NFκB was connected to more concepts in the original CRPS network than TP53.

An automatic network analysis approach where a variety of nodes (for example all highly connected proteins in the PathwayAssist database, all nodes generated by the expanded network algorithm, or simply all nodes in the PathwayAssist database) would be tested for positive association to a subnetwork of predefined disease-specific nodes would be valuable for both hypotheses generation and testing. Unfortunately, there is no such algorithm available in the PathwayAssist version used by the authors of this paper. Furthermore, the necessary data (specific connectivity information for each node in the database, i.e. which other nodes it is connected to) required for carrying out these types of tests outside the framework of the tool are not provided by PathwayAssist. The assessment of the connectivity to the CRPS network for NFκB and TP53 was performed manually inspection, an activity that is very time consuming and not defendable for a larger number of nodes. Despite the limitations regarding the assessment of the specificity of the results, a new node found by one of the network gap analysis algorithms provided by PathwayAssist, which was significantly related to the CRPS network, was considered important enough for further investigation.

One should bear in mind that results from text mining exercises as described in this paper are far from the solution to the medical problem or the complete answer to outstanding questions. The current tools in the biomedical domain are **not **capable of delivering clear and already assessed hypotheses. However, in this case, the results of a simple knowledge gap analysis provided a new idea that, after further manual exploration in literature, appeared very plausible and worthwhile investigating in biological experiments and epidemiological studies.

The generalizability of this method of hypothesis discovery needs to be assessed by repeating the exercise for other complex medical conditions. However, it would be an exhaustive and extremely time consuming task to repeat the exercise for a large group of complex disorders. This is a well-known problem in the evaluation of text mining tools in general [73].

### Testing the new hypothesis

New biological data are needed to verify the in silico derived hypothesis concerning the involvement of NFκB in the pathogenesis of CRPS. These data could be generated through animal models, clinical studies, and epidemiological studies. A rat model for CRPS has been developed by Coderre and colleagues [74]. They named it the chronic post ischemia pain (CPIP) model, since CRPS like symptoms are provoked by ischemia and reperfusion of the hindpaw by binding it temporarily with a tourniquet. This model may prove useful to investigate the role of NFκB in the early development of CRPS, for example by measuring the transcription of NFκB in the affected tissue. Additionally, in this model the effect of administration of NFκB inhibitors could be studied. In the past, NFκB knockout and transgenic animal models have been extensively used to study the NFκB pathway [75]. An increased inflammatory response coupled with an increased susceptibility to opportunistic infections has been recorded [76,77], and a decrease of the electroacupuncture-induced analgesic effects has also been shown [78]. A failure to induce CRPS like symptoms in NFκB knock mice would support the hypothesis of a NFκB as a crucial factor in CRPS.

Clinical and epidemiological studies to test the hypothesis could involve the investigation of determinants of altered NFκB activity and the comparison in this view between CRPS cases and healthy controls. For example it could be tested whether other NFκB related disorders (asthma, autoimmune disorders or atherosclerosis) co-occur with CRPS. Viral infections are known to upregulate NFκB activity. Thus the time relation between CRPS and the occurrence of a viral infection may also be worthwhile investigating. Finally, one could search for features from a genetic origin. The increased prevalence of an ACE polymorphism in CRPS patients was found in a small study [79]. Since ACE is also an inhibitor of NFκB activity, further investigations regarding this polymorphism might be of interest.

Certain drugs affect (perhaps unintentionally) the NFκB pathway and could influence the development of CRPS (see also section Targeting the NFκB pathway). Currently, according to the recently developed Dutch evidence based Guideline Complex Regional Pain Syndrome type 1 [80], nine drugs have been proven beneficial in the prevention or treatment of CRPS, including ketamine [81], gabapentine [82], DMSO crème [83], N-acetylcysteine [83], corticosteroids [84], bisfosfonates [85], calcium antagonists [86], ketanserine [87], and vitamin C [88]. Interestingly, 126 small molecules are listed as NFκB inhibitors in PathwayAssist, and 5 of these overlap with the list of drugs proven beneficial for preventing or treating CRPS (ketamine [89], DMSO crème [90], N-acetylcysteine [91], corticosteroids [92], and calcium antagonists [93]).

A Dutch cohort of CRPS patients was identified in the Integrated Primary Care Database (IPCI), a database that makes electronic patient records used in routine care available to investigators [94]. This cohort has been used before in research on CRPS [95] and will be used for further testing of these hypotheses within the framework of INFOBIOMED.

### Clinical relevance

NFκB is a transcription factor that is known to be involved in many processes, but its function is best described in inflammation [46]. In the recent past NFκB has been discovered as an important mediator in diseases due to chronic and exaggerated inflammatory responses, including sepsis, asthma, rheumatic disorders, inflammatory bowel disease, and psoriasis. However, in the current available literature NFκB has never before been mentioned in association with CRPS. Therefore, this relation is an interesting new result derived from the text mining exercise. Especially remarkable is that, in the generated network, NFκB is not only linked with the inflammatory concepts related in CRPS, but also to the non-inflammatory concepts in CRPS, such as neuropeptides and catecholamines.

**Figure 3:**
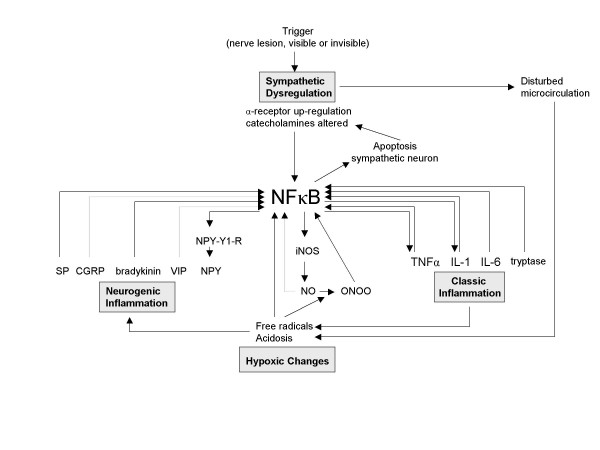
**The entire CRPS pathogenesis. Starting point: sympathetic dysregulation**. Mediated by NFκB, a trigger that induces sympathetic dysregulation can induce the entire pathogenesis of CRPS. SP = Substance P, CGRP = Calcitonin Gene-related Protein, VIP = Vasoactive Intestinal Protein, NPY = Neuropeptide Y, NPY-Y1-R = Neuropeptide Y-Y1 Receptor, NEP = Neutral-Endopeptidase, ACE = Angiotensin Converting Enzyme, SMP = Sympathetically Maintained Pain, NO = Nitric Oxide, iNOS = inducible Nitric Oxidase, ONOO = peroxynitrite

**Figure 4:**
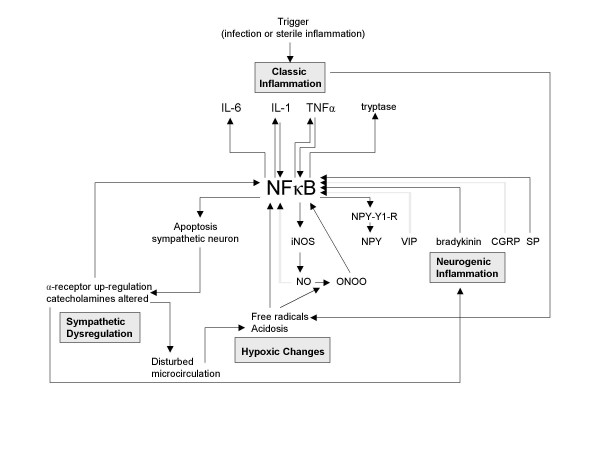
**The entire CRPS pathogenesis. Starting point: classic inflammation**. Mediated by NFκB, a trigger that induces classic inflammation can induce the entire pathogenesis of CRPS. SP = Substance P, CGRP = Calcitonin Gene-related Protein, VIP = Vasoactive Intestinal Protein, NPY = Neuropeptide Y, NPY-Y1-R = Neuropeptide Y-Y1 Receptor, NEP = Neutral-Endopeptidase, ACE = Angiotensin Converting Enzyme, SMP = Sympathetically Maintained Pain, NO = Nitric Oxide, iNOS = inducible Nitric Oxidase, ONOO = peroxynitrite

**Figure 5:**
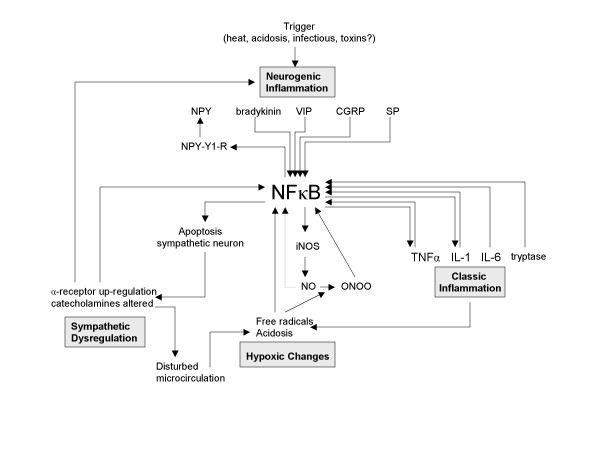
**The entire CRPS pathogenesis. Starting point: neurogenic inflammation**. Mediated by NFκB, a trigger that induces neurogenic inflammation can induce the entire pathogenesis of CRPS. SP = Substance P, CGRP = Calcitonin Gene-related Protein, VIP = Vasoactive Intestinal Protein, NPY = Neuropeptide Y, NPY-Y1-R = Neuropeptide Y-Y1 Receptor, NEP = Neutral-Endopeptidase, ACE = Angiotensin Converting Enzyme, SMP = Sympathetically Maintained Pain, NO = Nitric Oxide, iNOS = inducible Nitric Oxidase, ONOO = peroxynitrite

**Figure 6:**
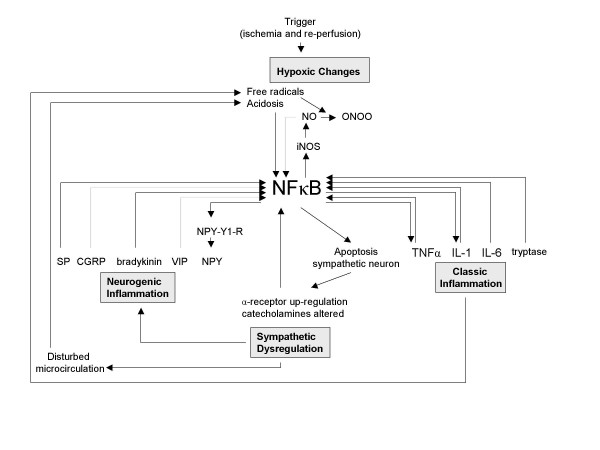
**The entire CRPS pathogenesis. Starting point: hypoxic changes**. Mediated by NFκB, a trigger that induces hypoxic changes can induce the entire pathogenesis of CRPS. SP = Substance P, CGRP = Calcitonin Gene-related Protein, VIP = Vasoactive Intestinal Protein, NPY = Neuropeptide Y, NPY-Y1-R = Neuropeptide Y-Y1 Receptor, NEP = Neutral-Endopeptidase, ACE = Angiotensin Converting Enzyme, SMP = Sympathetically Maintained Pain, NO = Nitric Oxide, iNOS = inducible Nitric Oxidase, ONOO = peroxynitrite

Realizing the possible central role for the NFκB pathway in the mechanisms underlying CRPS (or in the stress response caused by CRPS), unanswered questions concerning the disorder can be reviewed from a new point of view. For instance, when placing NFκB in the centre of the pathogenic process, the wide variety of precipitating events could be explained by the observation that all pathogenic mechanisms in CRPS are related to each other. This relation suggests that an event that specifically triggers one of the four mechanisms induces the entire complex pathogenic process (figure 3, figure 4, figure 5, and figure 6). For each mechanism separately the potential triggers are limited, but for the four mechanisms together the possibilities are numerous. With the addition of NFκB, the biochemical picture underlying CRPS becomes one in which several processes can initiate the disorder and in which NFκB can play a key role in its propagation.

Assuming the central unifying role of NFκB in the pathogenesis of CRPS, independent whether NFκB is the final molecule in the pathway or not, new targets for drug therapy could be provided. Current therapy in CRPS is aimed at targeting separate mechanisms. However, targeting one mechanism in CRPS is not enough, as made apparent by the limited success rate for the majority of treatments. Based on the new hypothesis, targeting the NFκB pathway may provide a way to target all the underlying mechanisms at once. Thereby, progression of the disorder may be more effective, since it will be prevented at more than one level. Independent from the inciting event or principle disturbed mechanism, NFκB pathway inhibitors might remove the 'engine' that keeps the process running.

Targeting NFκB as a therapy in sepsis, inflammatory diseases and neuronal diseases is proposed by several authors [47,50,96,97]. It is suggested to be very promising for a wide variety of patients, but due to the involvement of NFκB in a wide variety of physiological processes, there are safety implications. New drugs that target one of the components of the NFκB pathway are currently under development. One of these drugs, bortezomib, was already launched in the United States in 2003 for the treatment of multiple myeloma. Other drugs are in various stages of development (table 3). The NFκB pathway inhibitors are designed for the use in treatment of a variety of disorders, including inflammatory diseases, autoimmune diseases, atopic disorders, arteriosclerosis and malignancies. None of the drugs currently under development is mentioned for treatment of CRPS. When these drugs have proven to be safe, the therapeutic effect of NFκB pathway inhibitors could be studied in CRPS patients.

**Table 3:** Current development status of NFκB pathway inhibitors. (Investigational Drugs DataBase, Nov 15 2005 [106], advanced search by activity field on Nuclear factor kappa B inhibitor)

**Development status**	**No. of drugs**
Discovery Research	22
Clinical (unspecified)	1
Phase I clinical trials	4
Phase II clinical trials	4
Phase III clinical trials	0
Pre-registered	0
Registered	0
Launched	1 bortezomib
Research tools	1
Suspended	0
Withdrawn	0
No development reported	4
Discontinued	4

## Conclusion

Computer-assisted literature analysis to support the generation of novel and testable hypotheses has previously been proven useful [5,6]. This study builds upon this research, and extends it by bringing together researchers and clinicians with a wide variety of backgrounds. An experimental exercise was performed in which a text-mining/entity relationship tool has been applied to systematically synthesize the knowledge in existing literature about a complex disorder, exemplified by CRPS. Within the created literature network, a simple knowledge gap analysis was performed, using node connectivity. This approach was essential in formulating the hypothesis that NFκB might be a key player molecule in the pathogenesis of CRPS. This example of multidisciplinary research illustrates how the collaborative efforts of investigators from different fields of expertise can demonstrate new directions for future biological and epidemiological research on a complex disease.

## Competing interests

The author(s) declare that they have no competing interests.

## Authors' contributions

KMH participated in the design of the study, performed the pathway analyses and helped to draft the manuscript. MM provided biomedical knowledge on CRPS and drafted the manuscript. AGJB provided the biomedical concepts as input for thepathway analysesand participated in the design of the study. MW helped in performing the pathway and literature analyses and participated in the design of the study. EMM, JM and MC participated actively in the discussions and advised on the interpretation of the results. SB and JL conceived of the study, and participated in its design and coordination. SB also helped to draft the manuscript. All authors read, commented and approved the final manuscript.
